# Use of the Pay-for-Performance Program in Reducing Sarcopenia Risk: A Nested Case–Control Study Among Patients with Type 2 Diabetes Mellitus

**DOI:** 10.3390/medicina62010161

**Published:** 2026-01-13

**Authors:** Hui-Ju Huang, Pin-Fan Chen, Chieh-Tsung Yen, Ming-Chi Lu, Wei-Jen Chen, Tzung-Yi Tsai

**Affiliations:** 1 Department of Nursing, Dalin Tzu Chi Hospital, Buddhist Tzu Chi Medical Foundation, Chiayi 62247, Taiwan; df778383@tzuchi.com.tw; 2 Department of Metabolism &Endocrinology, Dalin Tzu Chi Hospital, Buddhist Tzu Chi Medical Foundation, Chiayi 62247, Taiwan; dm926840@tzuchi.com.tw; 3 School of Medicine, Tzu Chi University, Hualien 97004, Taiwan; 4 Department of Neurology, Dalin Tzu Chi Hospital, Buddhist Tzu Chi Medical Foundation, Chiayi 62247, Taiwan; dm335164@tzuchi.com.tw; 5 Division of Allergy, Immunology and Rheumatology, Dalin Tzu Chi Hospital, Buddhist Tzu Chi Medical Foundation, Chiayi 62247, Taiwan; 6 Graduate Institute of Sports Science, National Taiwan Sport University, Taoyuan 33325, Taiwan; 7 School of Post-Baccalaureate Chinese Medicine, Tzu Chi University, Hualien 97004, Taiwan; 8 Center of Sports Medicine, Dalin Tzu Chi Hospital, Buddhist Tzu Chi Medical Foundation, Chiayi 62247, Taiwan; 9 Department of Medical Research, Dalin Tzu Chi Hospital, Buddhist Tzu Chi Medical Foundation, Chiayi 62247, Taiwan; 10 Department of Environmental and Occupational Health, College of Medicine, National Cheng Kung University, Tainan 70428, Taiwan

**Keywords:** type 2 diabetes mellitus, pay-for-performance, sarcopenia, nested case–control study, risk

## Abstract

*Background and Objectives*: Despite substantial advances in treatment strategies for patients with type 2 diabetes mellitus (T2DM), its complication, particularly sarcopenia, has emerged as a global healthcare challenge. Pay-for-performance (P4P), an incentive-based payment scheme, has long been used to improve the quality of care; however, few studies have explored its effect on sarcopenia prevention. Therefore, we conducted a nested case–control study to investigate the association between P4P participation and the risk of sarcopenia among patients with T2DM. *Materials and Methods*: Using a large claims dataset, we identified individuals aged 20–70 years with newly diagnosed T2DM between 2001 and 2010 in Taiwan. All enrollees were followed up until 2013 to determine the occurrence of sarcopenia. For each case, we randomly matched two controls without sarcopenia. The risk of sarcopenia in relation to P4P participation was estimated by fitting conditional logistic regression to yield the adjusted odds ratio (aOR) and corresponding 95% confidence interval (CI). *Results*: A total of 3475 individuals with sarcopenia and 6948 matched controls were included. Patients enrolled in the P4P program had a lower risk of sarcopenia than their matched counterparts (aOR = 0.66; 95% CI: 0.61–0.74). Earlier P4P enrollment (within 1 year of T2DM diagnosis) and high-intensity P4P participation were associated with additional reductions in sarcopenia risk. *Conclusions*: Integrating P4P into routine T2DM care may help prevent sarcopenia, highlighting the importance of interdisciplinary collaboration and timely program implementation.

## 1. Introduction

Although advances in treatment strategies have increased life expectancy, chronic illnesses remain prevalent due to innate chronic inflammatory responses [1]. For example, the global prevalence of type 2 diabetes mellitus (T2DM) is estimated to increase from 5.9% in 2021 to 9.5% in 2050, representing an increase of more than 60% [2]. The growing incidence of diabetes has gradually placed healthcare systems on the verge of a disaster. In the United States, approximately US$307 billion was spent on the treatment and prevention of diabetes and its complications in 2022, and total societal costs may reach $413 billion after accounting for indirect expenses [3]. On average, the per capita cost burden associated with T2DM is nearly three-fold higher than that of individuals without diabetes ($19,736 vs. $7714) [4].

In addition to its substantial economic burden, the persistent hyperglycemia associated with T2DM may lead to skeletal muscle dysfunction, particularly sarcopenia [5]. A recent systematic review reported a significant association between T2DM and sarcopenia, with the prevalence of sarcopenia among patients with T2DM ranging from 6.3% to 47.13% [5]. Patients who develop sarcopenia have an 89% higher risk of cardiovascular disease than those with T2DM alone [6]. Although the detailed pathogenesis of sarcopenia in T2DMpopulations is not yet fully understood, the presence of advanced glycation end products (AGEs) resulting from hyperglycemia may trigger mitochondrial dysfunction, leading to the overproduction of reactive oxygen species and impaired energy production, both of which hinder the repair and regeneration of muscle fibers [7]. In addition, hyperglycemia is thought to promote inflammation by activating immune cells such as macrophages in adipose tissues, thereby stimulating circulating cytokines and inflammation-associated pathways [8]. The presence of pro-inflammatory profiles, such as interleukin (IL)-1β, IL-6, and tumor necrosis factor-α, can gradually impair muscle regenerative capacity by reducing satellite cell activity [7]. Based on this evidence, early efforts to prevent or delay the development of sarcopenia should be emphasized in routine diabetic care.

In general, intensive glycemic control is considered essential for slowing the progression of diabetes-related comorbidities. However, poor glycemic control remains a critical challenge in the management of diabetes. A cross-sectional study of 1253 adults with T2DM found that only one in five participants met the criteria for optimal glycemic control, defined as hemoglobin A1c (HbAlc) levels below 7% [8]. In response, pay-for-performance (P4P) programs for patients with high-cost and high-risk chronic diseases, such as diabetes, chronic kidney disease, and cancer, have gained considerable attention in recent years [9]. The P4P program, also known as value-based payment, aims to establish a comprehensive patient-centered care encompassing disease treatment, sequential health education, consultation, and structural referral systems [10]. By linking financial incentives to desired care quality and outcomes, healthcare providers are encouraged to implement coordinated care programs to enhance patient engagement in treatment plans and follow-up care [11]. Accumulating evidence indicates that integrating P4P into routine diabetic care reduces the burden of complications, such as infection rate and mortality risk [12,13], and improves the quality and satisfaction of healthcare services [14,15]. Despite this progress, no clear conclusion has been reached regarding the association between P4P participation and sarcopenia risk. Therefore, we conducted a population-based study to compare the risk of sarcopenia among patients with T2DM who did and did not receive P4P services. In addition to addressing this research gap, our findings may help inform the development of appropriate health policies and tailored programs for patients with T2DM.

## 2. Methods

### 2.1. Data Source

Data from the National Health Insurance (NHI) Database between 2000 and 2013 were used in this retrospective cohort-based case–control study. All deposited data were randomly sampled from nationwide health claims managed by the Health and Welfare Data Science Center of Taiwan. This database contains beneficiary information, such as sex, birth date, and physician billing records for comprehensive outpatient and inpatient visits covered by the NHI program [16]. By leveraging this dataset, researchers can generate critical insights into healthcare service utilization. All personal identifiers and healthcare utilization data were de-identified and spatially aggregated, enabling longitudinal follow-up. All medical diagnoses were recorded using the International Classification of Diseases, Ninth Revision, Clinical Modification (ICD-9-CM).

### 2.2. Ethics Statement

This study was conducted in accordance with the principles of the World Medical Association’s Declaration of Helsinki. Patient consent was waived due to the retrospective nature of the study and the exclusive use of anonymized data. (No. B10802014).

### 2.3. Identification of the Underlying Cohort

We first searched the database for individuals who met the threshold of at least three outpatient visits or at least one hospitalization with a T2DM diagnosis between 2001 and 2010 (ICD-9-CM code 250). The first date of seeking medical care was defined as the index date for T2DM onset. We then excluded individuals with incomplete baseline data on age or sex (n = 132), and those with a follow-up time of less than one year (n = 53). In addition, individuals with a history of sarcopenia before cohort entry were excluded to ensure the correct temporal sequence (n = 1163). A total of 66,269 patients with new-onset T2DM aged 20–70 years were included in the subsequent analysis.

### 2.4. Patient and Control Groups

**Figure 1:**
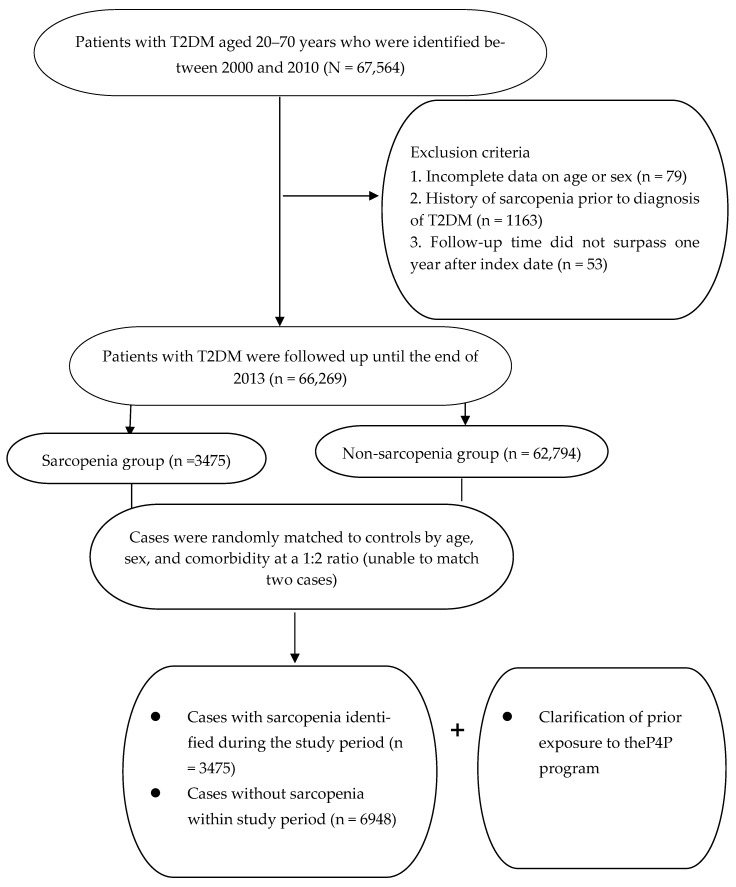
Flowchart of patient selection and screening for the study cohort.

The primary outcome was the occurrence of sarcopenia during the study period (2002–2013). Participants were classified as having sarcopenia if they had at least three outpatient clinic visits within 1 year or at least one hospitalization attributable to ICD-9-CM code 728.2 or 728.9 [17]. The index date was defined as the date of the first medical visit related to sarcopenia. Controls without sarcopenia were randomly selected from the remaining eligible individuals in the database at a 1:2 ratio, matched by age (year of birth), sociodemographic characteristics, and coexisting medical conditions, as assessed by the Charlson–Deyo Comorbidity Index (CCI) (Figure 1). Each control participant was assigned the same index date as the corresponding sarcopenia case to ensure comparable observation time for observing P4P exposure. Both cohorts were followed until the occurrence of a study endpoint or the end of 2013.

### 2.5. Clarification of P4P Exposure

Following a series of data extraction and linkage procedures, all recruited participants were matched to ambulatory claims to ascertain their exposure to the P4P program. Participants were classified as P4P users if any of their claims carried the notation “E4” during the period from cohort entry to the index date [18]. Individuals without such documentation were categorized as non-P4P users.

In Taiwan, P4P programs have long been implemented to provide integrated, multidisciplinary care for patients with complex or chronic conditions [9]. In 2021, the NHI Administration introduced the diabetic-specific P4P program to enhance the quality of care for patients with diabetes. Broadly, P4P represents a patient-tailored care model emphasizing structured assessment, individualized care planning, coordinated clinical management, and continuous follow-up, thus facilitating active patient engagement. To rigorously evaluate the association between P4P utilization and sarcopenia prevention, we quantified the total number of P4P visits throughout study period and classified it into three tertiles (low, middle, and high).

### 2.6. Definition of Covariates

Covariates incorporated into the statistical model included sex, age, prior medical comorbidities, monthly salary, and the urbanization level of the residential area. The monthly salary was inferred from health insurance premiums and categorized into three quartiles, with level 1 representing the lowest-income group. Residential districts were classified into three urbanization levels according to an established methodology that accounts for multiple demographic and socioeconomic factors, including population density per square kilometer, proportion of individuals with at least a bachelor’s degree, percentage of residents aged ≥65 years, proportion of the labor force engaged in agriculture, and number of clinicians per 100,000 residents [19]. This classification serves as a proxy for healthcare accessibility. Medical comorbidities were identified from diagnostic records that indicated at least one inpatient claim or two outpatient claims occurring within 1 year before cohort entry. Comorbidity burden was quantified using the CCI, which assigns weighted scores to 17 chronic conditions; higher scores reflect greater disease severity and a potentially increased impact on overall health status [20].

### 2.7. Data Analysis

All statistical analyses were performed using the standard software package (SAS for Windows, Version 9.4). Descriptive statistics, including means with standard deviations, frequencies, and percentages, were used to summarize baseline characteristics of the study population. Group differences between treated and control participants were evaluated using Student’s *t*-test for continuous variables and chi-square tests for categorical variables. To examine the association between P4P participation and the subsequent sarcopenia risk, conditional logistic regression was applied to the matched case–control dataset to estimate odds ratio (OR) and 95% confidence interval (CI). Adjusted OR (aOR) would be further calculated to control for the covariates listed in Table 1. Statistical significance was determined using two-tailed tests with an alpha level of ≤0.05; *p*-values below this threshold were considered statistically significant.

## 3. Results

Of the 10,423 patients with T2DM recruited for analysis, 3475 were newly diagnosed with sarcopenia, and 6948 were randomly matched controls without sarcopenia (Figure 1). The mean age of the enrollees was 55.7 ± 9.7 years. During the study period, 20.7% of cases and 27.6% of controls ever received the add-on P4P service. Overall, after the random matching process, the two groups were balanced with respect to the covariates assessed, thereby ensuring comparability before data analysis (Table 1).

**Figure 2:**
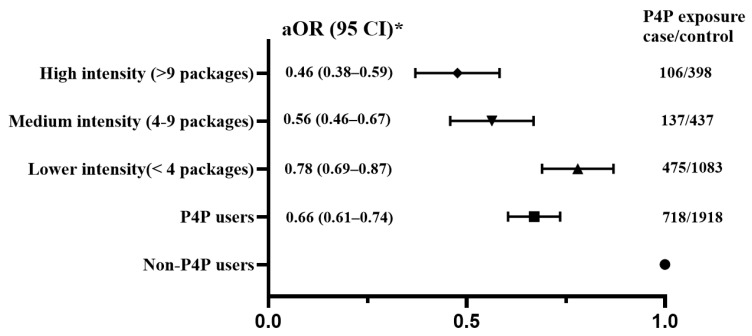
Association between sarcopenia risk and exposure to the P4P program. * Adjusted for potential confounders, including age, sex, residential area, monthly income, and CCI.

After fitting the conditional logistic regression model and adjusting for relevant covariates, we found that patients who received P4P care alongside conventional management had a lower risk of sarcopenia than those who did not (aOR = 0.66; 95% CI: 0.61–0.74). Subgroup analysis showed that the frequency of P4P use was significantly correlated with sarcopenia risk, demonstrating a clearly inverse exposure–response relationship (Table 2). Specifically, the aOR were 0.78 (95% CI: 0.69–0.87), 0.56 (95% CI: 0.46–0.67), and 0.46 (95% CI: 0.38–0.59) for low, medium, and high P4P intensities, respectively (Figure 2). The beneficial effect of P4P participation on preventing sarcopenia remained consistent forage and sex subgroup analysis (Table 2). Importantly, the benefit of P4P use in lowering sarcopenia risk gradually decreased with the delayed commencement of P4P program (Table 3).

## 4. Discussion

Currently, mounting evidence indicates that T2DM itself may incite the development of sarcopenia through the accumulation of AGEs, which in turn endangers cellular function and triggers apoptosis, ultimately impairing skeletal muscle mass and strength [5,6]. As there are no specific therapies for preventing sarcopenia after T2DM onset, identifying an adjunctive care strategy may offer promising insights for clinical practice [21]. The main objective of this study was to examine whether adding the P4P program to routine care could have a spillover effect on the prevention of progressive functional decline, especially in sarcopenia. We found that care combined with participation in the P4P program was significantly associated with a lower risk of sarcopenia. Moreover, the intensity of P4P participation was inversely correlated with sarcopenia risk. Participants receiving the high-intensity P4P program were strongly associated with a sarcopenia risk reduction of up to 54% compared with the matched comparators. In addition, early enrollment within 1 year after T2DM onset was markedly associated with a lower risk of sarcopenia. Although direct comparisons with matched relatives of target patients are scarce, the association observed herein aligns with prior studies and contributes to the growing body of evidence on this topic [11,12,13].

Several factors may account for the benefit of P4P in lowering sarcopenia risk. First, unlike conventional care, the P4P program aims to deliver tailored care to patients with diabetes through dynamic, collaborative, and highly interactive approaches [22]. Highly interactive strategies, including the use of colored images in P4P, have been reported to motivate patients with diabetes to acquire appropriate health information and assist them in building effective, personalized therapy plans after discharge [23]. Regular follow-up care embedded in the P4P program may also contribute to this benefit. A previous study revealed that participants in the P4P program had a 4.27-fold higher likelihood of receiving continuity of care than non-participants [24]. Regular follow-ups can strengthen patient–physician relationships, thereby enabling patients to adhere more effectively to treatment plans throughout the care process [25]. Furthermore, the improved adherence facilitated by P4P participation may encourage patients with diabetes to achieve tighter glycemic control, thus reducing inflammatory mediators triggered by hyperglycemia [8,24,26]. Prolonged hyperglycemia may lead to aberrant activation of inflammatory cascades, such as NF-kappaB and p38-MAPK pathways [27,28,29], resulting in the over-expression of molecules that induce satellite cell atrophy and impair cellular recovery [7]. As to age-and sex-specific analyses, it demonstrated that the P4P program provides greater benefits for young adults and females. Two possible explanations may account for these findings. First, prior studies have indicated that younger generations often possess more positive health consciousness than older populations, implying greater focus on wellness, regular exercise, and mental health [30]. Second, the pronounced benefit among females may be related to hormonal influences, particularly estrogen, which has been shown to suppress plasma concentrations of inflammatory mediators [31], thereby enhancing the efficacy of the P4P program.

Despite the important public health implications of this study, it has several limitations that should be acknowledged. First, the claims-based database in this study did not capture relevant information such as physical activity, psychosocial factors, dietary intake, or biomedical data. The absence of these variables may limit the precision of risk estimates. Future studies are warranted to validate our findings and address these gaps. Nevertheless, the applications of random matching combined with multivariate analyses likely balanced the two groups with respect to potential confounders, thereby ensuring comparability during data analyses. Moreover, given the magnitude and statistical significance of the effects observed in this large-scale survey, these limitations are unlikely to materially affect the primary findings. Second, reliance on secondary healthcare databases may have resulted in the accidental inclusion of miscoded patients, potentially introducing misclassification bias. To mitigate this concern, we restricted the analysis to individuals with newly diagnosed T2DM or sarcopenia who had at least three outpatient visits with consistent diagnoses or at least one inpatient admission. Additionally, the Taiwan NHI randomly reviews charts and audits medical charges, which reinforces the reliability of the claims data. Furthermore, because coding practices and data availability were comparable within both groups, any misclassification bias is likely to be non-differential, tending to attenuate associations rather than inflate effect estimates. Third, although the P4P program was associated with substantial benefits, caution is warranted in interpreting these results because participants were not randomly assigned to treatment and control groups at baseline. Future studies employing random assignment in ethnically diverse cohorts with T2DM are needed to provide more robust causal inference. Despite these limitations, the strength of this study lies in its large population-based dataset, which minimizes selection bias and enables stratified subgroup analyses to robustly examine the impact of P4P care after controlling for baseline covariates.

## 5. Conclusions

Given the absence of a definitive cure for sarcopenia in patients with T2DM, the most effective strategy is to quickly identify potential interventions that can prevent this condition. The present study supports the integration of the P4P program into routine care, demonstrating its association with a reduced risk of sarcopenia, particularly among patients receiving high-intensity P4P care (up to 54%). Moreover, early enrollment in the P4P program—especially within 1 year of T2DM onset—was strongly associated with a lower risk of sarcopenia. These findings not only help bridge existing knowledge gaps but also offer an insight to guide tailored healthcare services. In clinical practice, healthcare practitioners should provide educational resources that empower patients to manage diabetes-related symptoms, including musculoskeletal complications, thereby promoting better self-care capability and adaptation. Additionally, healthcare providers may regularly monitor physical performance among patients with diabetes using safe and cost-effective assessments. The innovative care strategies embedded in the P4P program are urgently needed to enhance patient outcomes and reduce the burden of sarcopenia.

## Figures and Tables

**Table 1:** Demographic characteristics and selected comorbidities of participants.

Variables	Total Group N = 10,423 (%)	Cases	Controls	*p*
		N = 3475 (%)	N = 6948 (%)	
Age (years)				0.55
≤50	3220 (30.9)	1087 (31.3)	2133 (30.7)	
>50	7203 (69.1)	2388 (68.7)	4815 (69.3)	
Mean (SD)	55.6 (9.7)	55.4 (9.8)	55.7 (9.6)	0.14
Sex				0.99
Female	6610 (57.7)	2004 (57.7)	4006 (57.7)	
Male	4413 (42.3)	1471 (33.3)	2942 (42.3)	
Monthly income				0.89
Low	3765 (36.1)	1256 (36.1)	2509 (36.1)	
Median	6139 (58.9)	2041 (58.7)	4098 (59.0)	
High	519 (5.0)	178 (5.1)	341 (4.9)	
Residential area				0.27
Urban	5466 (52.5)	1861 (53.6)	3605 (51.9)	
Suburban	1537 (14.7)	498 (14.3)	1039 (15.0)	
Rural	3420 (32.8)	1116 (32.1)	2304 (33.2)	
CCI	6.5 (8.5)	6.3 (8.1)	6.5 (8.9)	0.26

**Table 2:** Age- and sex-specific risk of sarcopenia in participants with and without P4P exposure.

Variables	Participants, n (%)	Crude OR (95% CI)	Adjusted OR * (95% CI)
Female			
Non-P4P users	1564 (78.0)	1	1
P4P users	440 (22.0)	0.66 (0.58–0.74)	0.65 (0.57–0.74) *
Male			
Non-P4P users	1193 (81.1)	1	1
P4P users	278 (18.9)	0.72 (0.61–0.83)	0.71 (0.61–0.83) *
Age ≤ 50			
Non-P4P users	845 (77.2)	1	1
P4P users	242 (22.3)	0.63 (0.53–0.74)	0.62 (0.53–0.73) **
Age > 50			
Non-P4P users	1912 (80.1)	1	1
P4P users	476 (19.9)	0.71 (0.62–0.79)	0.70 (0.61–0.79) **

* Model adjusted for age, urbanization level, monthly income, and CCI. ** Model adjusted for sex, urbanization level, monthly income, and CCI.

**Table 3:** Risk of sarcopenia among patients with T2DM according to adoption of the P4P program, stratified by timing of P4P initiation.

Follow-Up Duration	Non-P4P Users		P4P Users	
	Adjusted OR	95% CI	Adjusted OR ^a^	95% CI
<1 year	1	reference	0.41	0.30–0.57
1–3 years	1	reference	0.65	0.53–0.79
>3 years	1	reference	0.90	0.76–1.05

^a^ Model adjusted for age, sex, urbanization level, monthly income, and CCI.

## Data Availability

Due to legal restrictions imposed by the government in relation to the “Personal Information Protection Act,” data cannot be made publicly. The relevant data in this study are available on request from the corresponding author.
